# Deep learning-based solution for smart contract vulnerabilities detection

**DOI:** 10.1038/s41598-023-47219-0

**Published:** 2023-11-16

**Authors:** Xueyan Tang, Yuying Du, Alan Lai, Ze Zhang, Lingzhi Shi

**Affiliations:** Salus Security, Beijing, 100020 China

**Keywords:** Computational science, Computer science, Information technology, Software

## Abstract

This paper aims to explore the application of deep learning in smart contract vulnerabilities detection. Smart contracts are an essential part of blockchain technology and are crucial for developing decentralized applications. However, smart contract vulnerabilities can cause financial losses and system crashes. Static analysis tools are frequently used to detect vulnerabilities in smart contracts, but they often result in false positives and false negatives because of their high reliance on predefined rules and lack of semantic analysis capabilities. Furthermore, these predefined rules quickly become obsolete and fail to adapt or generalize to new data. In contrast, deep learning methods do not require predefined detection rules and can learn the features of vulnerabilities during the training process. In this paper, we introduce a solution called Lightning Cat which is based on deep learning techniques. We train three deep learning models for detecting vulnerabilities in smart contract: Optimized-CodeBERT, Optimized-LSTM, and Optimized-CNN. Experimental results show that, in the Lightning Cat we propose, Optimized-CodeBERT model surpasses other methods, achieving an f1-score of 93.53%. To precisely extract vulnerability features, we acquire segments of vulnerable code functions to retain critical vulnerability features. Using the CodeBERT pre-training model for data preprocessing, we could capture the syntax and semantics of the code more accurately. To demonstrate the feasibility of our proposed solution, we evaluate its performance using the SolidiFI-benchmark dataset, which consists of 9369 vulnerable contracts injected with vulnerabilities from seven different types.

## Introduction

Blockchain is a new application pattern based on technologies such as point-to-point transmission, encryption algorithm, consensus mechanism^1^ and distributed data storage^2^. Since the emergence of Bitcoin, the basic blockchain system has become widely known among professionals, resulting in the development of numerous blockchain applications. This is made possible by smart contracts^3^, which are automated programs running in a trusted environment provided by the blockchain^4^. If there are vulnerabilities in smart contracts publicly deployed on the blockchain, attackers can exploit these vulnerabilities to launch attacks. For example, on June 17, 2016, The DAO^5^, the largest crowdfunding project in the blockchain industry at the time, was attacked. The hacker exploited a reentrancy vulnerability and stole 3.6 million Ether worth around $60 million from The DAO’s asset pool, which directly led to the Ethereum blockchain splitting into ETH (Ethereum) and ETC (Ethereum Classic). On April 22, 2018, hackers targeted the BEC^6^ smart contract, which was based on the ERC-20 standard, using an integer overflow vulnerability. They transferred a significant amount of BEC tokens to two external accounts and dumped them, causing the token price to rapidly plummet to zero, disrupting the market. On February 15, 2020, the bZx protocol^7^, a set of smart contracts built on Ethereum, experienced its first attack. The attacker profited over three hundred thousand dollars, leading the project to temporarily suspend all functions except lending. On the 18th, the bZx protocol was targeted again, and the hacker exploited the manipulation of virtual asset prices through controlling the oracle, resulting in a profit of over 2,000 Ether. The above smart contract attack incidents demonstrate that due to the control of a substantial amount of cryptocurrency and financial assets, if smart contracts are targeted and attacked, it will result in unpredictable asset losses. Conducting vulnerability detection on smart contracts can help identify and fix potential vulnerabilities in contracts at an early stage, ensuring their security and protecting against asset theft or other security risks. Therefore, smart contract vulnerability detection is crucial for ensuring security, preventing financial losses, and maintaining user trust. It is an essential aspect of smart contract development and deployment processes.

Current methods for detecting smart contract vulnerabilities include human review, static analysis^8^, fuzz testing^9^, and formal verification^10^. Well-known detection tools include Oyente^11^, Mythril^12^, Securify^13^, Slither^14^, and Smartcheck^15^. These tools automatically analyze contract code and cover various common types of smart contract security vulnerabilities, such as reentrancy, incorrect tx.origin authorization, timestamp dependency, and unhandled exceptions. However, they may produce false positives or false negatives because they highly depend on predefined detection rules and lack the ability to accurately comprehend complex code logic. Additionally, predefined rules become outdated quickly and cannot adapt or generalize to new data, which is rapidly evolving in the smart contract domain. In contrast, deep learning approaches learn from data and can continuously update themselves to stay relevant. In recent years, there has been significant research on using deep learning for smart contract vulnerability detection^16–19^. However, some methods tend to overlook critical vulnerability features in their data processing approaches, and certain models lack semantic analysis capabilities for vulnerability code, leading to potential false negatives^20–22^.

This paper proposes a deep learning-based solution called Lightning Cat. The solution includes three deep learning models, namely optimized CodeBERT, Optimized-LSTM, and Optimized-CNN, which are trained to detect vulnerabilities in smart contracts. To better identify vulnerability features, code snippets of functions containing vulnerabilities were obtained to preserve key features. The CodeBERT pre-training model^23^ was employed to preprocess the data, enhancing the semantic analysis capabilities^24^.

The main contributions of this paper can be summarized as follows:

1. This paper designs a smart contracts vulnerabilities detection solution called Lightning Cat using deep learning methods. The solution optimizes three deep learning models.
2. We introduce an effective data preprocessing method that captures the semantic features of smart contract vulnerabilities. During the data preprocessing stage, we retrieve code snippets of functions containing vulnerabilities to extract vulnerability features. We also use the CodeBERT pre-trained model for data preprocessing to enhance the model’s semantic analysis capabilities, with the primary goal of improving model performance.
3. Based on the experimental evaluation results, the Lightning Cat proposed in this paper shows better detection performance than other vulnerability detection tools. The optimized CodeBERT model in Lightning Cat outperforms Optimized-LSTM and Optimized-CNN models, achieving a recall rate of 93.55%, which is 11.85% higher than Slither, a precision rate of 96.77%, and an f1-score of 93.53%.

In addition to smart contract vulnerability detection, the Lightning Cat can also be extended to other areas of code vulnerability detection^25^. Modern software systems are prone to various types of vulnerabilities, such as buffer overflow, null pointer dereference, and logic errors. For instance, buffer overflow code vulnerabilities are characterized by the use of unsafe string manipulation functions like strcpy and strcat without proper input boundary checks. Null pointer dereference vulnerabilities involve the misuse of dangling pointers by failing to set a pointer to NULL after freeing the associated memory. Logic errors manifest when incorrect logical operators are used in conditional statements, such as using = instead of ==. By learning and training on a large number of code samples, Lightning Cat can extract and comprehend different types of vulnerabilities. It also employs the CodeBERT pre-trained model for data preprocessing, making it better suited for identifying code vulnerabilities. As a result, it can detect various kinds of code vulnerabilities, thereby enhancing the security and dependability of the code.

## Related work

In this section, we present related work on the detection of smart contract vulnerabilities, focusing primarily on static analysis techniques and deep learning methods.

### Static analysis techniques

The Mythril^12^ security analysis method is designed to inspect bytecode executed in the Ethereum Virtual Machine (EVM). When defects are found in a program, it can help infer potential causes by analyzing input records. This assists in identifying existing vulnerabilities and reducing the likelihood of exploiting them. It utilizes taint analysis and symbolic execution techniques. However, when performing taint analysis, It faces limitations when crossing memory boundaries. This limitation becomes more severe when dealing with reference-style parameter invocations. Additionally, Mythril may encounter the state explosion problem when processing complex contracts. Furthermore, symbolic execution is a powerful general method for detecting vulnerabilities, but it may consider branches that may not be feasible in actual execution, leading to false positives.

Slither^14^ is a static code analysis tool used for detecting security vulnerabilities and potential issues in Solidity smart contracts. It integrates numerous detectors capable of identifying different types of vulnerabilities. Compared to Mythril, Slither is much more efficient and performs fast detection. However, Slither lacks formal semantic analysis, which limits its ability to perform more detailed security analysis and accurately determine low-level information such as gas calculations.

SmartCheck^15^ uses static analysis techniques to detect common security vulnerabilities and coding issues in smart contracts. It offers numerous rule sets to identify different types of vulnerabilities and improve contract security. However, due to its heavy reliance on logical rules for vulnerability detection, it may generate false positives and false negatives. Furthermore, it may fail to detect severe programming errors, leading to overlooked vulnerabilities or incorrect reporting.

### Pre-training model

The analysis of smart contracts using deep learning methods in our study is essentially a Natural Language Processing (NLP)^26^ task. In general NLP tasks, the input texts are required to be represented as vectors which can be further fed into the deep learning models for downstream tasks. With the development of pre-trained models, the text representation in NLP has presented a significant improvement. Pre-trained models can effectively capture the semantic information, textual structure and grammar rules as well as provide valuable vector embeddings via training on large-scale datasets. They are relatively crucial in NLP. In this research, we employ a pre-trained model CodeBERT in transforming smart contracts code based on text into vector embeddings. A distinctive feature of CodeBERT is that it has been pre-trained on a vast amount of code and its associated natural language comments, which enables it to understand the structure and semantics of the code.

CodeBERT, as a pre-trained model, presents state-of-the-art performances on both natural language processing and programming language processing tasks as downstream tasks. The characteristic of CodeBERT is that it has been pre-trained on a vast amount of code and its associated natural language comments, enabling it to understand the structure and semantics of the code. Typical pre-trained models like Seq2Seq, Transformer, and RoBERTa generally perform well in PL-NL processing tasks. Furthermore, CodeBERT has a gain of around 3.5, 2, and 1 BLEU^27^ score over Seq2Seq^28^, Transformer^28^, and RoBERTa^29^ models in code-to-documentation generation tasks^23^, which means CodeBERT possesses better programming language processing ability.

### Deep learning for smart contract vulnerabilities detection

From related work, it has been observed that some tools based on static analysis techniques suffer from false positives and false negatives, mainly due to their reliance on predefined rules. These tools lack the ability to perform syntax and semantic analysis, and the predefined rules can become outdated quickly and cannot adapt or generalize to new data. In contrast, deep learning methods do not require predefined detection rules and can learn vulnerability features during the training process.

We have found that some literature has utilized deep learning for smart contract vulnerabilities detection. Zhipeng Gao^30^ and his team developed a deep learning tool that analyzes Solidity smart contracts on the Ethereum blockchain, helping developers detect repetitive patterns and known bugs. Zhang et al.^18^ proposed a Novel Smart Contract Vulnerability Detection Method Based on Information Graph and Ensemble Learning. In the same year, they also introduced the Serial-Parallel Convolutional Bidirectional Gated Recurrent Network Model incorporating Ensemble Classifiers (SPCBIG-EC) for enhanced smart contract vulnerability detection in IoT devices^19^.

Huang et al.^20^ proposed a vulnerabilities detection model for smart contracts using a convolutional neural network. This network converts the binary representation of vulnerable code into RGB images. However, converting binary files to image format makes it challenging to preserve the syntax and semantic information of the code. Although this approach improves accuracy to some extent^31^, it suffers from a high false negative rate.

Liao et al.^21^ used N-gram language modeling and tf-idf feature vectors to characterize smart contract source code. They trained traditional machine learning models to verify 13 types of vulnerabilities and employed a gray-box fuzz testing mechanism for real-time transaction validation. However, this method treats certain critical opcodes as stop words during the representation process, which can result in false negatives and missed detections.

Yu et al.^22^ developed the first systematic and modular framework for smart contract vulnerability detection based on deep learning. They introduced the concept of Vulnerability Candidate Slice (VCS), which focuses on analyzing the dependencies between diverse data and control program elements. Experimental results showed a significant improvement of 25.76% in the F1 score using this approach. However, the performance improvement is not substantial for vulnerability types with limited data and control flow dependencies.

These works provide various methods for data preprocessing aimed at enabling the deep learning models to extract vulnerability features more effectively. However, some methods may result in the deletion of important keywords or the ignoring of critical vulnerability features during data processing^32^. Additionally, some of the models used may have an insufficient understanding of the semantic characteristics of vulnerability code programs^33^, which can result in false negatives. To address these issues, we utilized the CodeBERT pre-training model for data preprocessing. CodeBERT is a Transformer-based pre-training model designed specifically for learning and processing source code. It demonstrates stronger semantic analysis abilities, providing significant advantages in smart contract vulnerability detection. Additionally, we introduced the concept of critical vulnerability code segments and removed code unrelated to vulnerabilities from the training samples. We retained only the function code of critical vulnerabilities for learning. This strategy eliminates training noise introduced by redundant code, reduces model complexity, and improves model performance. During the model training stage, we utilized three models - optimized CodeBERT, Optimized-LSTM, and Optimized-CNN - to capture vulnerability features more effectively.

Using the aforementioned methods, our proposed Lightning Cat tool extracts critical features from vulnerability code and has strong semantic analysis capabilities, which significantly improves model performance.

## Methodology

CodeBERT model has the state-of-the-art performances in tasks related to programming language processing^23^. It features capturing semantic connections between natural language and programming language. According to Yuan et al.^34^, CodeBERT can achieve 61% of accuracy in software vulnerabilities discovery which is generally higher than mainstream models Word2Vec^35^, FastText^36^ and GloVe^37^ (46%, 41% and 29% respectively). In our research, smart contracts are based on programming language Solidity. Therefore, we optimize the CodeBERT model and employ it in our study. CNN is a commonly used and typical deep learning model with an excellent generality in processing images and texts. LSTM is also a deep learning model featuring in processing long texts and it can effectively learn time sequence in texts which CNN is not adaptive to do. Both CNN and LSTM have achieved significantly high accuracy (0.958 and 0.959 respectively) in source code vulnerabilities detection, according to Xu et al.^38^. We attempt to employ CNN and LSTM models as comparisons with CodeBERT model and further analyze the performances of them in our tasks.

**Figure 1:**
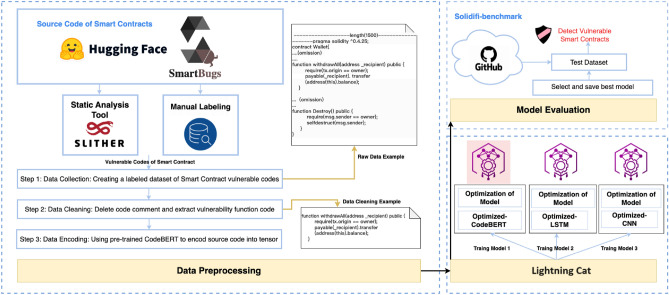
Lightning Cat Model Development Process.

Figure 1 illustrates the complete process of developing a vulnerability detection model called Lightning Cat for smart contracts, which consists of three stages. The first stage involves building and preprocessing the labeled dataset of vulnerable Solidity code. In the second stage, training three models (Optimized-CodeBERT, Optimized-LSTM, and Optimized-CNN) and comparing their performance to determine the best one. Finally, in the third stage, the selected model is evaluated using the Sodifi-benchmark dataset to assess its effectiveness in detecting vulnerabilities.

### Data preprocessing

During the data preprocessing phase, we collect three datasets and subsequently perform data cleaning. Finally, we employ the CodeBERT model to encode the data.

#### Data collection

Our primary training dataset comprises three main sources: 10,000 contracts from the Slither Audited Smart Contracts Dataset^39^, 20,000 contracts from smartbugs-wild^40^, and 1,000 typical smart contracts with vulnerabilities identified through expert audits, overall 31,000 contracts. To effectively compare results with other auditing tools, we choose to use the SolidiFI benchmark dataset^41^ as our test set, a dataset containing contracts containing 9,369 bugs.

#### Dataset processing

Within our test set SolidiFI-benchmark, there are three static detection tools which are Slither, Mythril, and Smatcheck as well as all identified four common vulnerability types which are Re-entrancy, Timestamp-Dependency, Unhandled-Exception, and tx.origin. To ensure the completeness and fairness of the results, our proposed Lightning Cat model primarily focused on these four types of vulnerabilities for comparison. Table 1 displays the mapping between the four types of vulnerabilities and the three auditing tools.

**Table 1:** Mapping of Four Vulnerability Types.

Vulnerability	Slither	Smartcheck	Mythril
Re-entrancy	Reentrancy-benign reentrancy-eth reentrancy-unlimited-gas reentrancy-no-eth	SOLIDITY_ETRNANCY	External call to user-supplied address external call to fixed address state change after external call
Timestamp-dependency	Timestamp	SOLIDITY_EXACT_TIME VYPER_TIMESTAMP_DEPENDENCE	Dependence on predictable environment variable
Unhandled-exceptions	Unchecked-send unchecked-lowlevel	SOLIDITY_UNCHECKED_CALL	Unchecked call return value
tx.origin	tx-origin	SOLIDITY_TX_ORIGIN	Use of tx.origin

**Figure 2:**
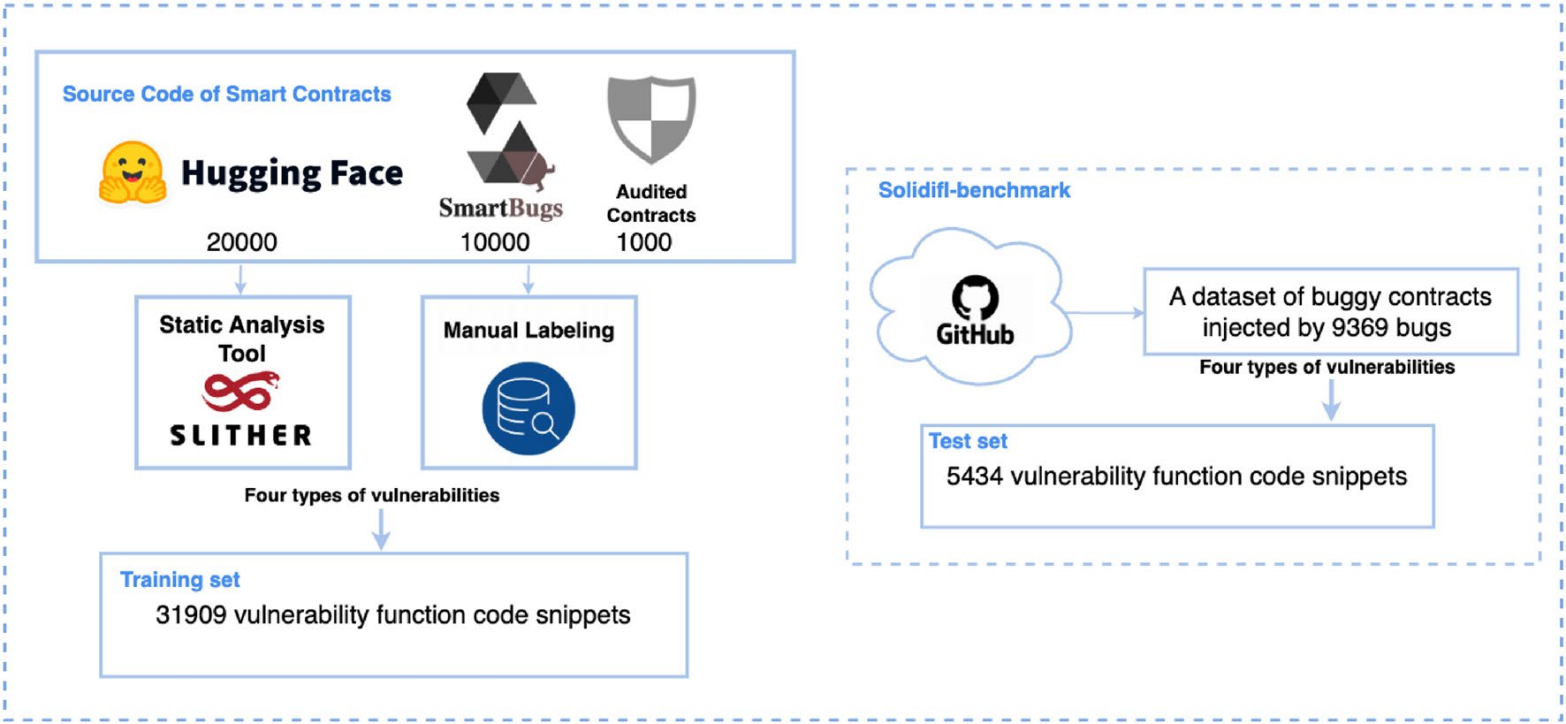
Datasets Preprocessing.

Considering that a complete contract might consist of multiple Solidity files and a single Solidity file might contain several vulnerable code snippets, we utilized the Slither tool to extract 30,000 functions containing these four types of vulnerabilities from the data sources^39,40^. Additionally, we manually annotate the problematic code snippets within the contracts audited by experts, overall 1,909 snippets. The training set comprises 31,909 code snippets. For the test set, we extract 5,434 code snippets related to these four vulnerabilities from the SolidiFI-benchmark dataset. The processing procedures for the training and test sets can be seen in Fig. 2.

#### Data cleaning

The length of a smart contract typically depends on its functionality and complexity. Some complex contracts can exceed several thousand tokens. However, handling long text has been a long-standing challenge in deep learning^42^. Transformer-based models can only handle a maximum of 512 tokens. Therefore, we attempted two methods to address the issue of text length exceeding 510 tokens.

##### Direct splitting

The data is split into chunks of 510 tokens each, and all the chunks are assigned the same label. For example, if we have a group of Re-entrancy vulnerability code with a length of 2000 tokens, it would be split into four chunks, each containing 510 tokens. If there are chunks with fewer than 510 tokens, we pad them with zeros. However, the training results show that the model’s loss does not converge. We speculate that this is due to the introduction of noise from unrelated chunks, which negatively affects the model’s generalization capability.

##### Vulnerability function code extraction

Audit experts extracted the function code of vulnerabilities from smart contracts and assigned corresponding vulnerability labels. If the extracted code exceeds 510 tokens, it is truncated, and if the code falls short of 510 tokens, it is padded with zeros. This approach ensures consistent input data length, addresses the length limitation of Transformer models, and preserves the characteristics of the vulnerabilities.

**Figure 3:**
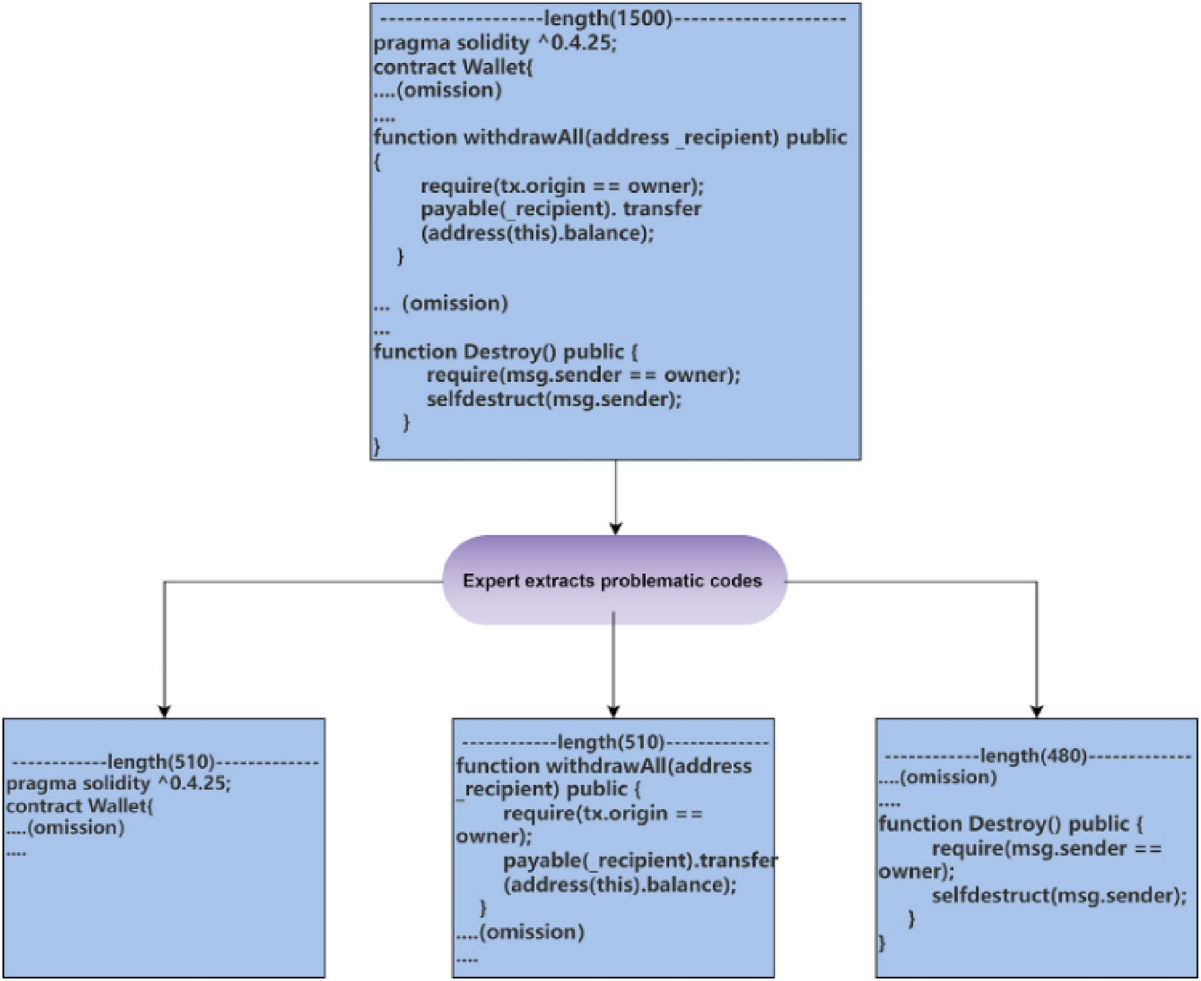
Extraction of Vulnerable Function Code (We partition the smart contract as a whole and extract only the functions where the vulnerabilities are present. In the provided image, we focus on the ”withdrawALL” function, which serves as our training dataset. If a contract contains multiple vulnerabilities, we extract multiple corresponding functions).

After comparing the two methods, we observed that training on vulnerability-based function code helped the model’s loss function converge better. Therefore, we chose to use this data processing method in subsequent experiments. Additionally, we removed unrelated characters such as comments and newline characters from the functions to enhance the model’s performance. As shown in Fig. 3, we only extracted the function parts containing the vulnerability code, reducing the length of the training dataset while maintaining the vulnerability characteristics. This approach not only improves the model’s accuracy, but also enhances its generalization ability.

#### Data encoding

CodeBERT is a pretraining model based on the Transformer architecture, specifically designed for learning and processing source code. By undergoing pretraining on extensive code corpora, CodeBERT acquires knowledge of the syntax and semantic relationships inherent in source code, as well as the interactive dynamics between different code segments.

During the data preprocessing stage, CodeBERT is employed due to its strong representation ability. The source code undergoes tokenization, where it is segmented into tokens that represent semantic units. Subsequently, the tokenized sequence is encoded into numerical representations, with each token mapped to a unique integer ID, forming the input token ID sequence. To meet the model’s input requirements, padding and truncation operations are applied, ensuring a fixed sequence length. Additionally, an attention mask is generated to distinguish relevant positions from padded positions containing invalid information. Thus, the processed data includes input IDs and attention masks, transforming the source code text into a numericalized format compatible with the model while indicating the relevant information through the attention mask.

For Optimized-LSTM and Optimized-CNN models, direct processing of input IDs and masks is not feasible. Therefore, CodeBERT is utilized to further process the data and convert it into tensor representations of embedding vectors. The input IDs and attention masks obtained from the preprocessing steps are passed to the CodeBERT model to obtain meaningful representations of the source code data. These embedding vectors can be used as inputs for Optimized-LSTM and Optimized-CNN models, facilitating their integration for subsequent vulnerability detection.

### Models

In the current stage, our approach involves the utilization of three machine learning models: Optimized-CodeBERT, Optimized-LSTM, and Optimized-CNN. The CodeBERT model is specifically fine-tuned to enhance its compatibility with the target task by accepting preprocessed input IDs and attention masks as input. However, in the case of Optimized-LSTM and Optimized-CNN models, we do not conduct any fine-tuning on the CodeBERT model for data preprocessing.

#### Model 1: optimized-CodeBERT

**Figure 4:**
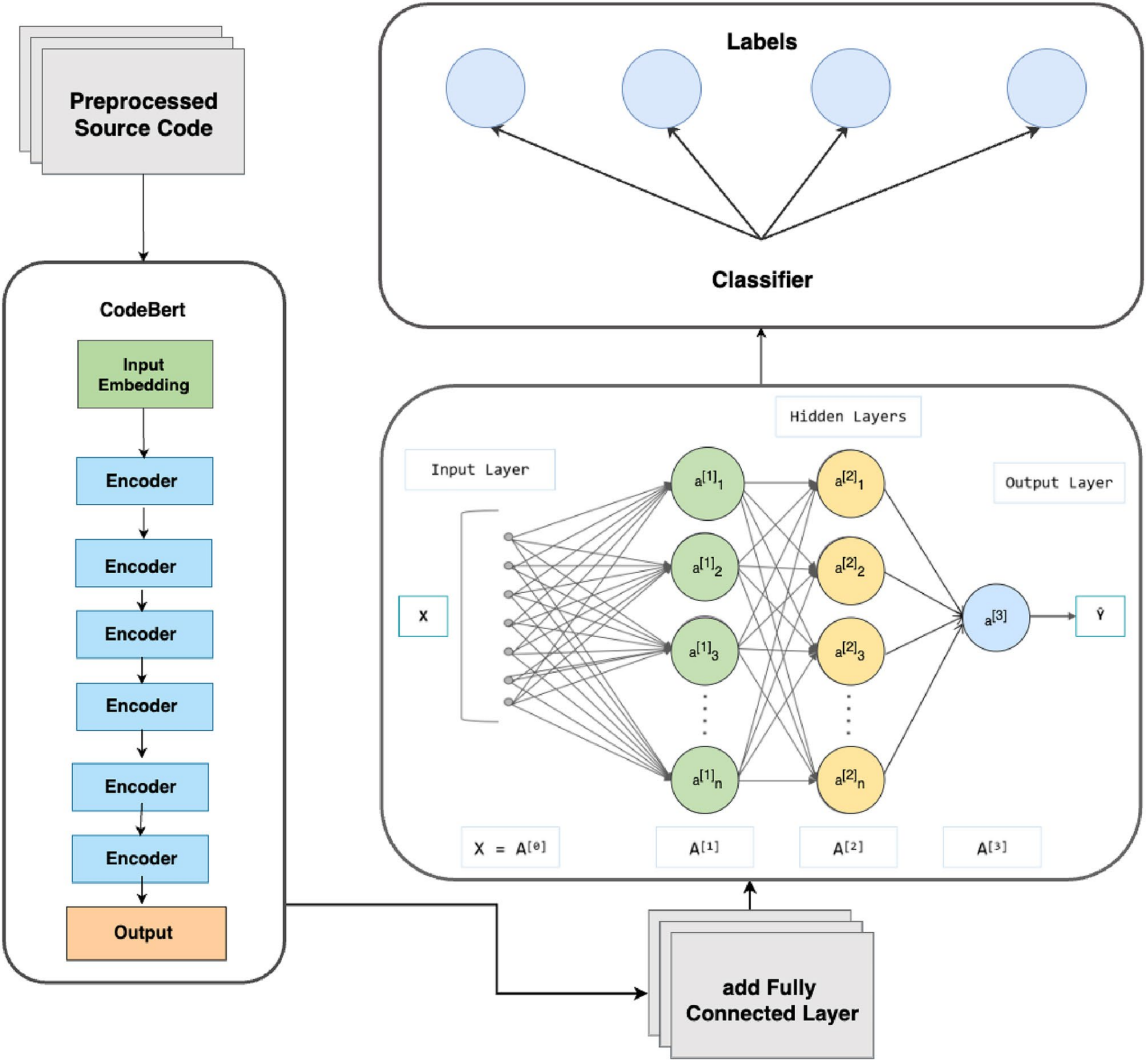
Our Optimized-CodeBERT Model Architecture.

CodeBERT is a specialized application that utilizes the Transformer model for learning code representations in code-related tasks. In this paper, we focus on fine-tuning the CodeBERT model to specifically address the needs of smart contract vulnerability detection. The CodeBERT model is built upon the Transformer architecture, which comprises multiple encoder layers. Prior to entering the encoder layers of CodeBERT, the input data undergoes an embedding process. Following the encoding stage of CodeBERT, fully connected layers are added for classification purposes. The model architecture of our CodeBERT implementation is depicted in Fig. 4.

Word Embedding and Position Encoding In the data preprocessing stage, we have utilized a specialized CodeBERT tokenizer to process each word into the input information. In this model tranining stage, the tokenizer employs embedding methods, which are used to convert text or symbol data into vector representations. This processing transforms each word into a 512-dimensional word embedding. In addition, we introduce position embedding, which is a technique introduced to assist the model in understanding the positional information within the sequence. It associates each position with a specific vector representation to express the relative positions of tokens in the sequence. For a given position *i* and dimension *k*, the Position Encoding $\text {PE}(i, k)$ is computed as follows:

$$\begin{aligned} \text {PE}(i, k) = {\left\{ \begin{array}{ll} \sin \left( \frac{i}{10000^{2k/d}}\right) &{} \text {if } k \text { is even} \\ \cos \left( \frac{i}{10000^{2k/d}}\right) &{} \text {if } k \text { is odd} \end{array}\right. } \end{aligned}$$

**Figure 5:**
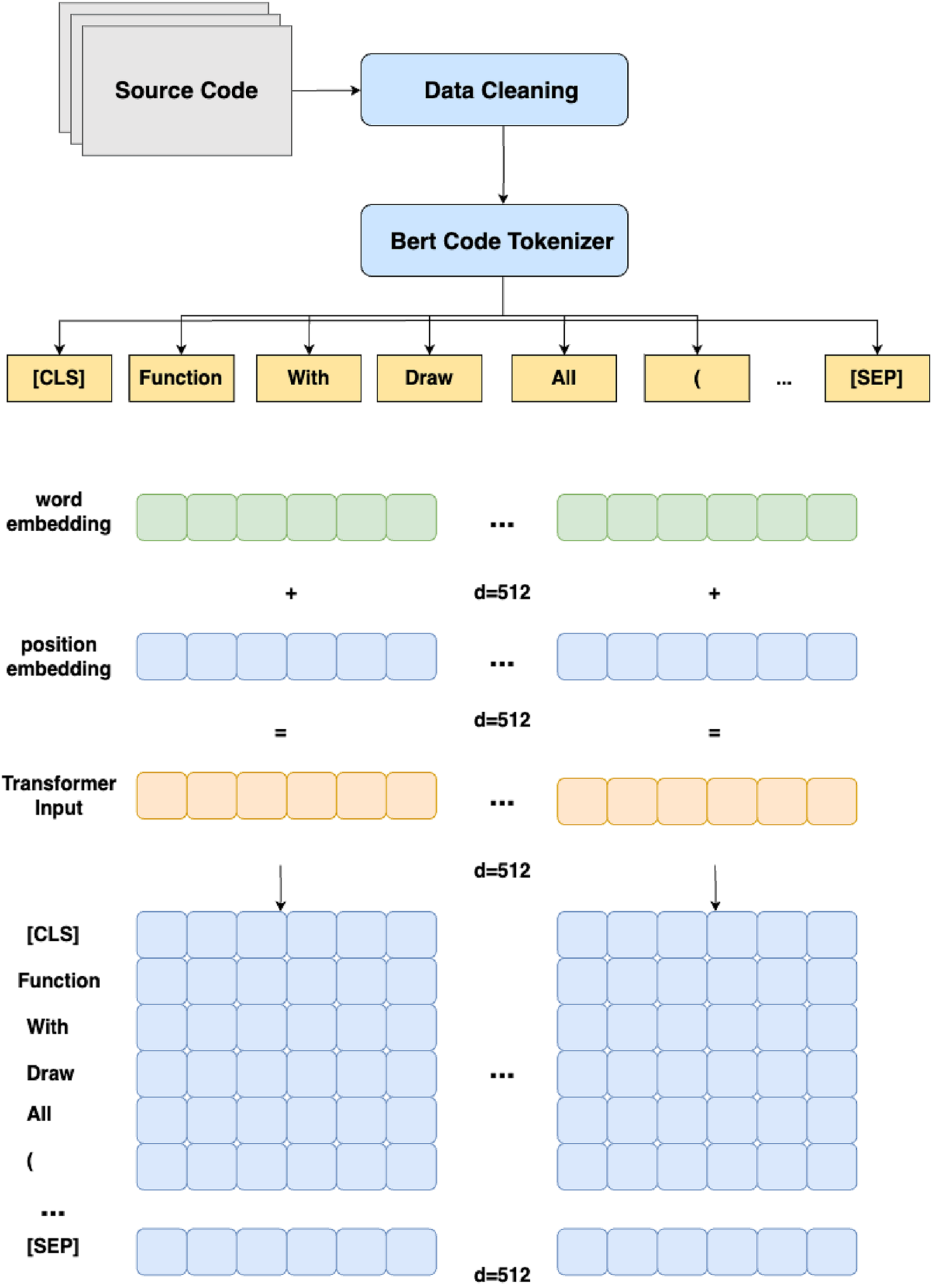
Word and Position Embedding Process.

    Here, *d* represents the dimension of the input sequence. The formula utilizes sine and cosine functions to generate position vectors, injecting positional information into the embeddings. The exponential term $\frac{i}{10000^{2k/d}}$ controls the rate of change of the position encoding, ensuring differentiation among positions. By adding the Position Encoding to the Word Embedding, positional information is integrated into the embedded representation of the input sequence. This enables CodeBERT to better comprehend the semantics and contextual relationships of different positions in the code. The processing steps are illustrated in Fig. 5.

Encoder layers The CodeBERT model performs deep representation learning by stacking multiple encoder layers. Each encoder layer comprises two sub-layers: multi-head self-attention and feed-forward neural network. The self-attention mechanism helps encode the relationships and dependencies between different positions in the input sequence. The feed-forward neural network is responsible for independently transforming and mapping the features at each position.

The multi-head self-attention mechanism calculates attention weights, denoted as $w_{ij}$, for each position *i* in the input code sequence. The attention weights are computed using the following equation:

$$\begin{aligned} w_{ij} = \text {Softmax}\left( \frac{{q_i \cdot k_j}}{\sqrt{d}}\right) \end{aligned}$$

    Here, $q_i$ represents the query at position *i*, $k_j$ denotes the key at position *j*, and *d* is the dimension of the queries and keys. The output of the self-attention mechanism at position *i*, denoted as $o_i$, is obtained by multiplying the attention weights $w_{ij}$ with their corresponding values $v_j$ and summing them up:

$$\begin{aligned} o_i = \sum _{j=1}^{n} w_{ij} \cdot v_j \end{aligned}$$

where *n* is the length of the input sequence.

Each encoder layer also contains a feed-forward neural network sub-layer, which processes the output of the self-attention sub-layer using the following equation:

$$\begin{aligned} \text {FFN}(x) = \text {ReLU}(x \cdot W_1 + b_1) \cdot W_2 + b_2 \end{aligned}$$

    Here, *x* represents the output of the self-attention sub-layer, and $W_1, b_1$ and $W_2, b_2$ are the parameters of the feed-forward neural network.

Fully connected layers To output the classification labels, we added fully connected layers. Firstly, we added a new linear layer with 100 features on top of the existing linear layer. To avoid the limited capacity of a single linear layer, we utilized the ReLU activation function. Additionally, to prevent overfitting, we introduced a dropout layer with a dropout rate of 0.1 after the activation layer. Lastly, we used a linear layer with four features for the output. During the fine-tuning process, the parameters of these new layers were updated.

#### Model 2: optimized-LSTM

**Figure 6:**
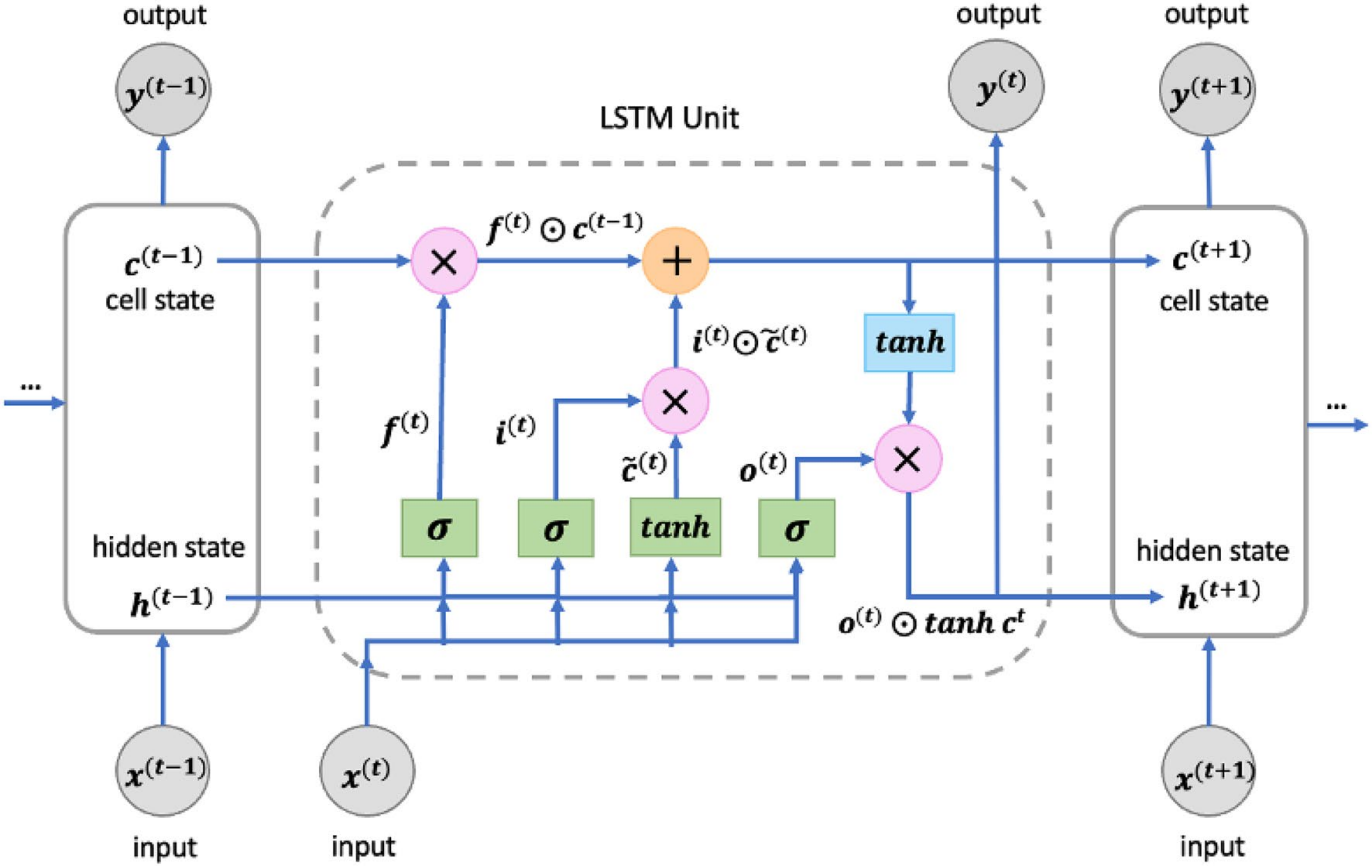
The Architecture of Optimized-LSTM.

The Optimized-LSTM model is specifically designed for processing sequential data, capable of capturing temporal dependencies and syntactic-semantic information^43^. For the task of smart contract vulnerability detection, our constructed Optimized-LSTM model provides a serialization-based representation of Solidity source code, taking into account the order of statements and function calls. The Optimized-LSTM model captures the syntax, semantics, and dependencies within the code, enabling an understanding of the logical structure and execution flow. Compared to traditional RNNs, the Optimized-LSTM model we constructed addresses the issue of vanishing or exploding gradients when handling long sequences^44^. This is accomplished through the key mechanism of gated cells, which enable selective retention or forgetting of previous states. The model consists of shared components across time steps, including the cell, input gate, output gate, and forget gate. In the Optimized-LSTM model, we have defined an LSTM layer and a fully connected layer, with the LSTM layer being the core component. Within the LSTM layer, the input $x^{(t)}$, the output from the previous time step $h^{(t-1)}$, and the cell state from the previous time step $c^{(t-1)}$ are fed into an LSTM unit. This unit contains a forget gate $f^{(t)}$, an input gate $i^{(t)}$, and an output gate $o^{(t)}$, as shown in Fig. 6.

In the model, we utilize a bidirectional Optimized-LSTM, where the forward Optimized-LSTM and backward Optimized-LSTM are independent and concatenated at the final step. This allows for better capture of long-term dependencies and local correlations within the sequence. During the forward propagation of the model, the input *x* is first passed through the Optimized-LSTM layer to obtain the output *h* and the final cell state *c*. Since the lengths of the data instances may vary, we calculate the average output by averaging the outputs at each time step in *h*. Then, the average output is fed into a fully connected layer to obtain the final prediction output *y*. We used the cross-entropy loss function *L* for training, which is defined as:

$$\begin{aligned} L_i=-\sum _{j=1}^N y_{i,j}\log {\hat{y}}_{i,j}. \end{aligned}$$

    Here, *N* represents the number of classes, $y_{(i,j)}$ denotes the probability of the *jth* class in the true label of sample *i*, and ${\hat{y}}_{(i,j)}$ represents the probability of sample *i* being predicted as the *jth* class by the model.

#### Model 3: optimized-CNN

The Convolutional Neural Network (CNN) is a feedforward neural network that exhibits remarkable advantages when processing two-dimensional data, such as the two-dimensional structures represented by code^45^. In our model design, we transform the code token sequence into a matrix, and CNN efficiently extracts local features of the code and captures the spatial structure, effectively capturing the syntax structure, relationships between code blocks, and important patterns within the code.

The Optimized-CNN primarily consists of convolutional layers, pooling layers, fully connected layers, and activation functions. Its core idea is to extract features from input data through convolution operations, reduce the dimensionality of feature maps through pooling layers, and ultimately perform classification or regression tasks through fully connected layers^46^. The key module of the Optimized-CNN is the convolutional layer, which is computed as follows:

$$\begin{aligned} y_{i,j}=\sigma \left( \sum _{k=1}^K\sum _{l=1}^L\sum _{m=1}^M w_{k,l,m}x_{i+l-1,j+m-1,k}+b\right) \end{aligned}$$

    Here, $x_{(i,j,k)}$ represents the element value of the input data at the *i*-th row, *j*-th column, and *k*-th channel, $w_{(k,l,m)}$ represents the weight value of the *k*-th channel, *l*-th row, and *m*-th column of the convolutional kernel, and *b* represents the bias term. $\sigma$ denotes the activation function, and in this case, we use the Rectified Linear Unit (ReLU).

The output of the convolutional layer is passed to the pooling layer for further processing. The commonly used pooling methods are Max Pooling and Average Pooling. In this case, we employ Max Pooling, and the calculation formula is as follows:

$$\begin{aligned} y_{i,j}=\max \limits _{m=1}^M\max \limits _{n=1}^N x_{i+m-1,j+n-1} \end{aligned}$$

    Pooling operations can reduce the dimensionality of feature maps, model parameters, and to some extent alleviate overfitting issues. Finally, a fully connected layer is used to compute the model, which is expressed as:

$$\begin{aligned} y=\sigma (Wx+b) \end{aligned}$$

**Figure 7:**
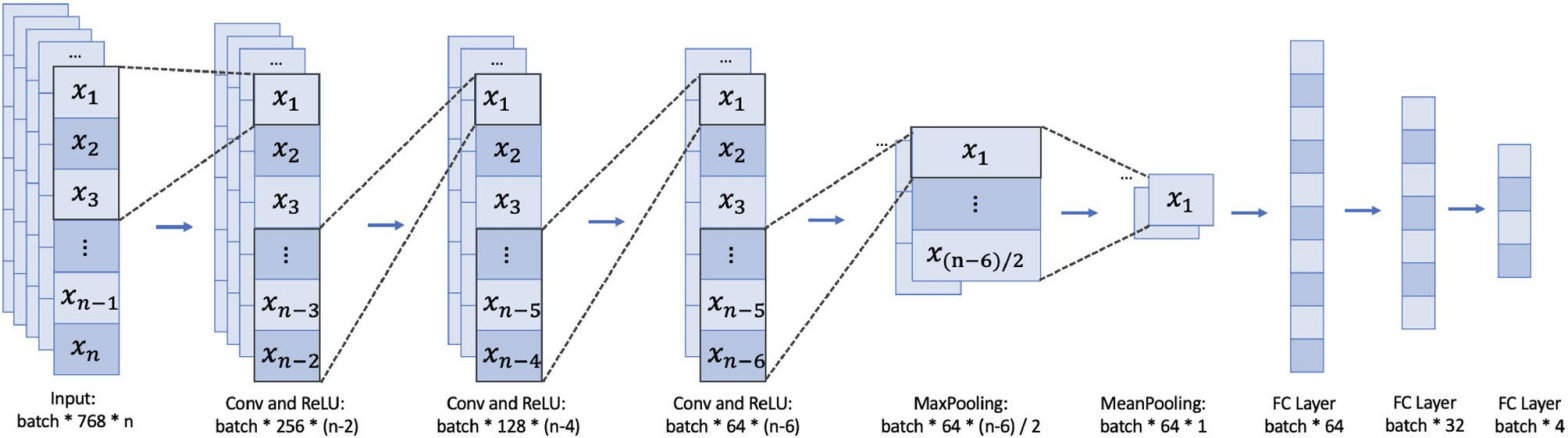
The Architecture of Optimized-CNN.

    Here, *x* represents the output of the previous layer, *W* and *b* denote the weights and bias terms, and $\sigma$ is the activation function. By stacking multiple convolutional layers, pooling layers, and fully connected layers, we construct a Optimized-CNN model as shown in Fig. 7, which has powerful feature extraction and classification capabilities for smart contract classification.

## Experiments

To ensure a fair evaluation of different methods, we trained and tested them in identical environments. All experiments were performed on a computer featuring an Intel Xeon(R) Silver 4210R CPU clocked at 2.4GHz, dual RTX A5000 GPUs, and 128GB of RAM, running on the Windows operating system, utilizing the PyCharm software, the PyTorch framework, and the Python programming language.

### Parameter settings

Then, we do the tuning process with respect to each hyperparameter. For Optimized-CodeBERT model, we employed the AdamW optimizer^47^ and conducted a grid search to find the optimal settings for hyperparameters. The hyperparameters and their corresponding search ranges were as follows: learning rate: (3e-5, 1e-4, 3e-4), batch size: (32, 64, 128, 256), dropout rate: (0.1, 0.2, 0.3, 0.4, 0.5), L2 regularization: (1e-6, 1e-5, 1e-4, 1e-3, 1e-2, 1e-1), learning rate decay gamma: (0.98, 0.99), number of fully connected layers: (1, 2, 3), and number of epochs: (10, 20, 30, 40, 50, 60). The cross-entropy loss was calculated using the BCEWithLogitsLoss method. The best parameter settings corresponding to the final results are shown in Table 2.

**Table 2:** Parameters of the Optimized-CodeBERT Model.

Model parameters	Configuration
Layer of MLP	2
Epoch	60
Batch size	128
Learning rate	1e − 3
Dropout	0.1
Optimizer	Adam
Loss function	BCEWithLogitsLoss
Learning rate decay gamma	0.98
L2 regularization	1e-4

For Optimized-LSTM Model, the best parameter settings are shown in Table 3.

**Table 3:** Parameters of the Optimized-LSTM Model.

Layer parameters	Configuration
Learning rate	0.0001
Input dimension	768
Hidden dimension	128
Number of layers	2
Bidirectional	True
Batch first	True
Dropout	0.5
Output dimension	4

For Optimized-CNN Model, the best parameter settings are shown in Table 4.

**Table 4:** Parameters of the Optimized-CNN Model.

Layer parameters	Configuration
Learning rate	0.0001
Conv1d_1	in_channels=768, out_channels=256, kernel_size=3
Conv1d_2	in_channels=256, out_channels=128, kernel_size=3
Conv1d_3	in_channels=128, out_channels=64, kernel_size=3
MaxPool1d	kernel_size=2
Linear_1	in_channels=64, out_channels=32
Linear_2	in_channels=32, out_channels=6

### Metrics

To evaluate our methods, we use various performance metrics, including accuracy, F1 score, recall, and precision. Accuracy is indeed the ratio of correctly predicted instances (both true positives and true negatives) to the total number of instances. It provides a general measure of overall correctness.

$$\begin{aligned} \text {Accuracy} = \frac{{\text {True Positives} + \text {True Negatives}}}{{\text {True Positives} + \text {True Negatives} + \text {False Positives} + \text {False Negatives}}} \end{aligned}$$

    F1 score is another important metric that combines both precision and recall. It considers the trade-off between them and provides a balance between the two. F1 score is particularly useful when the dataset is imbalanced or when both precision and recall are important.

$$\begin{aligned} \text {F1 score} = 2 \times \frac{{\text {Precision} \times \text {Recall}}}{{\text {Precision} + \text {Recall}}} \end{aligned}$$

    Precision, also known as positive predictive value, measures the proportion of correctly predicted positive instances (true positives) out of all instances predicted as positive. It focuses on the accuracy of positive predictions.

$$\begin{aligned} \text {Precision} = \frac{{\text {True Positives}}}{{\text {True Positives} + \text {False Positives}}} \end{aligned}$$

    Recall, also known as sensitivity or true positive rate, calculates the proportion of correctly predicted positive instances (true positives) out of all actual positive instances. It focuses on the ability of the model to identify all positive instances.

$$\begin{aligned} \text {Recall} = \frac{{\text {True Positives}}}{{\text {True Positives} + \text {False Negatives}}} \end{aligned}$$

### Results

We used SolidiFI-benchmark as the testing dataset, and the comparison results of the metrics of the three models in Lightning Cat are shown in Table 5.

**Table 5:** Comparison of Metrics Results of Three Models.

Metriccs	F1 (%)	Accuracy (%)	Precision (%)	Recall (%)
Optimized-CodeBERT	93.53	96.77	96.77	93.55
Optimized-LSTM	63.05	81.96	73.61	64.06
Optimized-CNN	70.62	85.54	71.61	71.36

Clearly, the performance of Optimized-CodeBERT is the best among all models (as shown in Table 5), with the highest scores in all metrics. Its F1-score is 30.48% higher than that of Optimized-LSTM and 22.91% higher than that of Optimized-CNN.

In addition, we obtained the metrics results of the three models for four types of vulnerabilities, as shown in Table 6.

**Table 6:** Comparison of the Metrics Results for Each Type of Vulnerability.

Vulnerability	Method	Accuracy(%)	Precision(%)	Recall(%)	F1(%)
Re-entrancy	Optimized-CodeBERT	93.58	85.45	89.20	87.29
	Optimized-LSTM	75.08	49.80	100	66.49
	Optimized-CNN	91	73.31	100	84.60
Timestamp-Dependency	Optimized-CodeBERT	97.29	90.43	99.92	94.94
	Optimized-LSTM	85.08	75.49	61.12	67.55
	Optimized-CNN	75.27	51.62	42.795	46.79
Unhandled-Exceptions	Optimized-CodeBERT	96.23	100	100	99.96
	Optimized-LSTM	84.63	100	39.23	56.35
	Optimized-CNN	80.53	61.52	61.43	61.47
tx.origin	Optimized-CodeBERT	99.98	99.93	85.08	91.94
	Optimized-LSTM	83.03	69.17	55.91	61.84
	Optimized-CNN	95.38	100	81.21	89.63

From Table 6, it can be seen that among the four types of vulnerabilities, Optimized-CodeBERT has the best detection performance for Timestamp-Dependency and Unhandled-Exceptions. However, for the Re-entrancy vulnerability, the Recall of Optimized-CodeBERT is lower than that of Optimized-LSTM and Optimized-CNN. When detecting the tx.origin vulnerability, Optimized-CodeBERT has higher Accuracy, Recall, and F1 than the other two models, while its Precision is 0.07% lower than that of Optimized-CNN. The three models use the same training set, but their detection performance differs because they have different modeling and generalization capabilities. Overall, Optimized-CodeBERT has better detection performance.

We obtained the vulnerability detection results of different methods in the SolidiFI-benchmark testing dataset, and the true positive results detected by each method are shown in Table 7. ”NA” indicates vulnerabilities that cannot be identified by the corresponding method.

**Table 7:** True Positive Detection Results of Different Methods.

Security bug	Re-entrancy	Timestamp dep	Unhandle exp	tx.origin
Injected bugs	1343	1381	1374	1336
Manticore	93	NA	NA	NA
Mythril	258	571	618	891
Oyente	335	0	322	NA
Securify	1111	NA	701	NA
Smartcheck	0	479	49	97
Slither	1343	844	917	1336
Optimized-CodeBERT	1198	1380	1169	1336
Optimized-LSTM	1343	844	539	747
Optimized-CNN	1343	591	844	1085

Table 7 displays the vulnerability detection results for different types of vulnerabilities. ”Injected bugs” represents the actual quantity of vulnerabilities, and ”tp” (true positive) represents the number of detected vulnerabilities. From Table 7, it can be seen that among these auditing tools (Manticore, Mythril, Oyente, Security, Slither), Slither detected the most actual vulnerabilities. In terms of the detection results of all methods, Slither, Optimized-LSTM, and Optimized-CNN detected the most true positive for the Re-entrancy vulnerability, while Optimized-CodeBERT detected the most vulnerabilities for the three types of vulnerabilities (Timestamp_Dependency, Unhandle_Exceptions, tx.origin).

**Figure 8:**
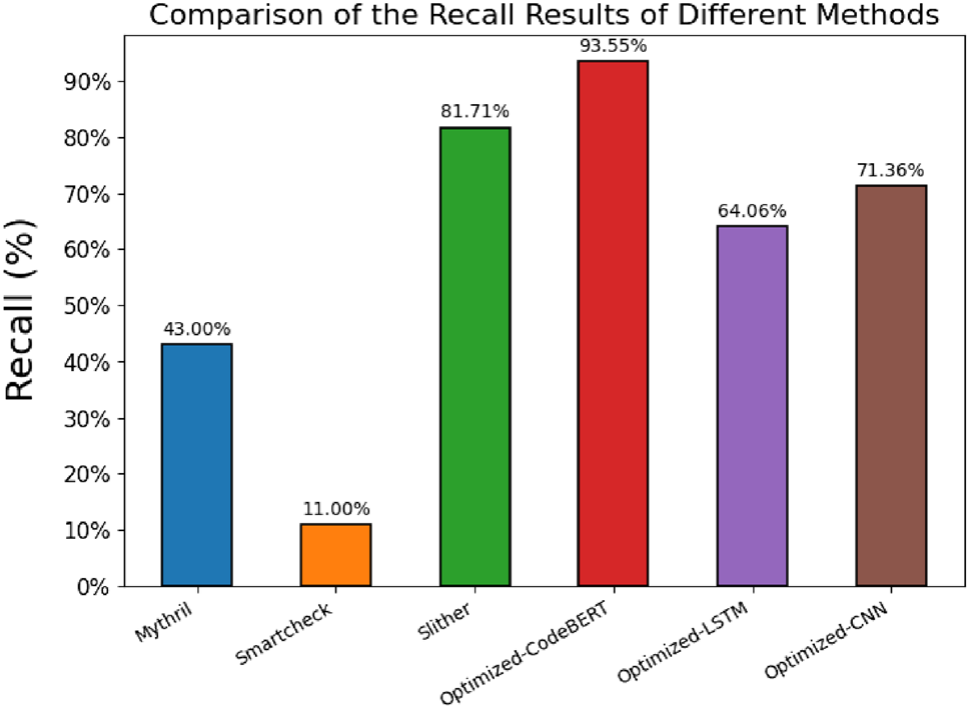
Enter Caption.

To better compare the detection performance of different methods, we will only compare the methods that can detect the four types of vulnerabilities. The comparison results of the Recall of different methods are shown in Fig. 8.

Figure 8 illustrates the comparison of Recall results among different methods, measuring the classification models’ capability to accurately identify true positive samples. The figure compares the recall rates of six methods, namely Mythril, Smartcheck, Slither, Optimized-CodeBERT, Optimized-LSTM, and Optimized-CNN. The findings indicate that the Optimized-CodeBERT model exhibits the highest recall at 93.55%, surpassing Slither by 11.85%. This highlights the Optimized-CodeBERT model’s exceptional accuracy and reliability in detecting and identifying true positive samples. Conversely, the Optimized-LSTM and Optimized-CNN models demonstrate relatively lower recall rates of 64.06% and 71.36%, respectively, suggesting potential challenges or limitations in recognizing true positive samples.

Based on the insights gained from Fig. 8, it is evident that the Optimized-CodeBERT method excels in recall, displaying superior proficiency in identifying true positive samples. These findings offer valuable guidance for model selection and practical applications.

**Figure 9:**
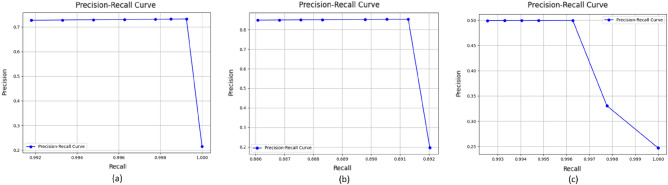
Precision-recall curves for three models :(**a**) is Optimised-CodeBERT, (**b**) is Optimised-CNN and (**c**) is Optimised-LSTM.

In order to comprehensively compare the performances of three models, we set different thresholds (i.e. 0.1, 0.2, 0.3, 0.4, 0.6, 0.7, 0.8, 0.9) for three models. The precision and recall values under these thresholds and precision-recall curves of three models are presented in the Fig. 9. The precision-recall curve is created by plotting precision values on the y-axis and recall values on the x-axis. Each point on the curve represents a different threshold used for classifying the positive class. Thresholds refer to the probability thresholds used by the classification model to determine which class an instance belongs to. According to the result shown in the Fig. 9, Optimised -CodeBERT presents a better performance in our tasks.

We further conduct McNemmar test^48^ to compare the accuracy differences between the Optimised-CodeBERT model and the Optimsed-LSTM (shown in Table 8) as well as Optimised-CNN models (shown in Table 9). Optimised-CodeBERT presents extreme significance in accuracy difference with both Optimised-LSTM and Optimised-CNN in terms of vulnerabilities classes Re-entrancy, Timestamp-Dependency and Unhandled-Exceptions but little significance with these two models in terms of vulnerability class tx.origin. We speculate that this is because the features of the tx.origin vulnerability are relatively conspicuous, making it easier for the model to recognize and classify.

**Table 8:** McNemmar Test (Accuracy Difference between Optimised-CodeBERT and Optimised-LSTM).

Vulnerability type	Discordant pairs (B)	Continuity correction (C)	Chi-squared value (${\mathscr {X}}^2$)	p-value	Accuracy difference
Re-entrancy	2638	1	1761.44	$\approx 0$	Extremely significant
Timestamp-dependency	147	1	285.33	$\approx 0$	Extremely significant
tx.origin	1	1	0	$\approx 1$	Not significant
Unhandled-exceptions	205	1	203.25	$\approx 0$	Extremely significant

**Table 9:** McNemmar Test (Accuracy Difference between Optimised-CodeBERT and Optimised-CNN).

Vulnerability type	Discordant pairs (B)	Continuity correction (C)	Chi-squared value (${\mathscr {X}}^2$)	p-value	Accuracy difference
Re-entrancy	489	1	377.33	$\approx 0$	Extremely significant
Timestamp-dependency	136	1	315	$\approx 0$	Extremely significant
tx.origin	1	1	0	$\approx 1$	Not significant
Unhandled-exceptions	530	1	1056	$\approx 0$	Extremely significant

### Discussion and future work

1. Scope of Vulnerability Detection and limitation.

In this research, we introduce a solution named ”Lightning Cat”. For the purpose of comparison with static analysis tools, we particularly focus on detecting four specific types of smart contract vulnerabilities: Re-entrancy, Timestamp-Dependency, Unhandled-Exceptions, and tx.origin. Through our comparative analysis, we observe instances where the static analysis tools fail to detect certain vulnerabilities. Our experimental results indicate that our proposed Optimized-CodeBERT outperforms the static detection tools mentioned in this paper, both in terms of recall and overall TP. At present, the Lightning Cat is equipped to detect these four categories of vulnerabilities.

While the proposed solution, which captures vulnerability feature information by extracting vulnerability code functions, has shown promising results in scenarios with text length constraints, we also acknowledge limitations in our current approach when addressing cross-code and cross-function vulnerabilities. The code interactions and contextual information related to these vulnerabilities might span multiple functions or modules, making them challenging to be captured by our current method. In the future, We aim to expand the detection capabilities of our model. Additionally, there’s potential to extend the application of Lightning Cat to other vulnerability code detection scenarios.

Q2: Expanding Lightning Cat to Other Areas of Code Vulnerability Detection.

Lightning Cat plans to expand its vulnerability detection capabilities. Using examples of vulnerabilities such as Buffer Overflow, Null Pointer Dereference, and Logic Errors, here’s how the expansion process unfolds:

Acquiring datasets containing code with these vulnerability features is the first step, which can be obtained from open-source code repositories or proprietary databases. Code mutation techniques may also be considered for data augmentation. During data preprocessing, CodeBERT will be used to convert code into fixed-length vectors suitable for the model’s input format. Subsequently, the model undergoes fine-tuning through transfer learning, with iterative adjustments to hyperparameters based on dataset characteristics to enhance performance. Validation using appropriate vulnerability test sets ensures model accuracy and robustness. Finally, to address newly emerging vulnerabilities, Lightning Cat periodically collects new vulnerability data and updates model parameters to effectively respond to these new issues.

Q3: What if malicious actors use this technology to discover vulnerabilities for illicit gain?

Automatic vulnerability detection technology holds immense potential in enhancing the security of smart contracts. However, it is a double-edged sword. On the one hand, it can assist developers in swiftly identifying and rectifying vulnerabilities in software, thereby elevating its security. On the other hand, if such technology falls into the hands of malicious actors, they might exploit it to uncover undisclosed vulnerabilities and launch attacks. To address this, proactive measures are essential.

Developers should regularly conduct code audits and undergo secure coding training as well as adopt responsible vulnerability disclosure policies. It’s encouraged that researchers and developers, upon discovering security vulnerabilities, initially notify the relevant organizations or individuals privately. This provides them ample time for rectification before the information is made public. Concurrently, regular updates and maintenance of software and dependency libraries are crucial to ensure known security vulnerabilities which are addressed collaboratively. While it might reduce the use of the tool, considerations could be made to impose certain restrictions on open-source automatic detection tools, such as limiting the use of advanced features or requiring user authentication. Undoubtedly, enhancing the security of smart contracts requires collective effort. The open-source community should foster collaboration among its members. This can be achieved by sharing best security practices, tools, and resources.

## Conclusion

This paper introduces Lightning Cat, which uses deep learning methods to detect vulnerabilities in smart contracts, including three models: Optimized-CodeBERT, Optimized-LSTM, and Optimized-CNN. Based on experimental results, the Optimized-CodeBERT model achieved the best overall performance. We optimized and compared three models, and found that Optimized-CodeBERT achieved the best results in evaluation metrics such as Accuracy, Precision, and F1-score. This research utilized the CodeBERT pre-trained model for data preprocessing, which improved the ability of code semantic analysis. In data preprocessing, we extracted problem code segments functions, which not only considered the key features of smart contract vulnerability code but also solved the length limitation problem of deep learning for processing long texts. This approach avoids issues such as unclear features due to excessively long texts or overfitting due to excessively short texts, thereby improving the model’s performance. The results show that the proposed method has more reasonable data preprocessing and model optimization, resulting in better detection performance.

This paper analyzed the detection performance of each type of vulnerability and found that the Optimized-CodeBERT model outperformed Slither, Optimized-LSTM, and Optimized-CNN in detecting three types of vulnerabilities, but was inferior in one type. This is because different models have different structures, parameters, and learning algorithms, which affect their modeling and generalization abilities. Therefore, in future work, we aim to improve the performance of the three models in Lightning Cat and extend the application of our proposed Lightning Cat to more code security fields beyond smart contract vulnerabilities detection (Supplementary Information).

### Supplementary Information

Supplementary Information.

## Data Availability

The datasets generated and/or analyzed during the current study are available from the corresponding author upon reasonable request.
