# Supplementary material for: Deep learning-based solution for smart contract vulnerabilities detection
Source: Sci Rep. 2023 Nov 16;13:20106. doi: 10.1038/s41598-023-47219-0 (PMC10654660; doi:10.1038/s41598-023-47219-0)
Supplement: Supplementary file 1 — Supplementary Information. [file 41598_2023_47219_MOESM1_ESM.pdf]

Supplementary Information for "Deep Learning-Based Solution for Smart Contract Vulnerabilities Detection"

Xueyan Tang[1*], Yuying Du[1], Alan Lai[1], Ze Zhang[1], and Lingzhi Shi[1]

[1]Salus Security, Beijing, 100020, China
[*]777728@gmail.com

We provide four examples of representative smart contract vulnerabilities, namely Reentrancy Vulnerabilities, Incorrect tx.origin Authorization, Timestamp Dependency, and Unhandled Exceptions.

(1) Reentrancy Vulnerabilities

Reentrancy vulnerability has always been the most harmful and frequently occurring type of vulnerability. It is related to external call and callback mechanism. The key is that when the victim contract calls the malicious contract fallback function for transfer, it will call back the victim contract itself, resulting in recursive calls, causing a loop transfer and illegal outflow of funds. Although the frequency of basic reentrancy vulnerabilities in smart contract code has decreased due to the increased security awareness of developers, they still cannot escape from different types of variant reentrancy attacks, such as Token-callback Reentrancy, Cross-function Reentrancy, Basic Reentrancy with a Twist, Cross-contract Reentrancy, and Read Only Reentrancy. Taking Read Only Reentrancy as an example, we will explain how the attack occurs.

```
function addLiquidity(uint256 eth_amount)
    external payable nonReentrant()
    returns (uint256)
{
    //Some necessary checks
    uint256 eth_reserve = address(this).balance - eth_amount;
    uint256 totalLiquidity = lpToken.totalSupply();
    uint256 lp_amount = (eth_amount * totalLiquidity) / eth_reserve;
    require(lp_amount > 0, "No Liquidity Shares Minted");
    lpToken.mint(msg.sender, lp_amount);
    return eth_amount;
}

function removeLiquidity(uint256 lp_amount)
    external nonReentrant()
    returns (uint256)
{
    //Some necessary checks
    uint256 eth_balance = address(this).balance;
    uint256 totalLiquidity = lpToken.totalSupply();
    uint256 eth_amount = (lp_amount * eth_balance) / totalLiquidity;
    (bool success, ) = msg.sender.call{value: eth_amount}("");
    require(success, "Transfer failed");
    lpToken.burn(msg.sender, lp_amount);
    return eth_amount;
}

function getSpotPriceEth(uint256 amount) public view returns (uint256)
{
    return amount * address(this).balance / lpToken.totalSupply();
}
```

Supplementary Figure 1. Example of Read Only Reentrancy

The location where the Read Only Reentrancy vulnerability occurs is the function in the smart contract that is marked as "view". This type of function does not change the state variables in the contract and is generally not decorated with mutex locks. When the victim contract calls the function marked as "view" in the vulnerable contract, it may cause abnormal conditions due to

obtaining the state that has not been updated in time, affecting subsequent operations.

As shown in the Supplement Figure 1, users can provide liquidity assets (using ETH as an example) to the pool to obtain the corresponding LP tokens, or remove their liquidity assets from the pool and get back the corresponding ETH. The calculation method for the LP tokens and ETH exchange ratio can be found in line 8 and line 21. There are three functions, addLiquidity, removeLiquidity, and getSpotPriceEth. The addLiquidity function exchanges ETH for LP tokens, and the removeLiquidity function exchanges LP tokens for ETH. Both of these functions have external visibility and inherit the nonreentrant function modifier from the OpenZeppelin official library. The getSpotPriceEth function is used to calculate the virtual value of LP tokens, i.e., the ETH share corresponding to a certain amount of LP tokens. This function is declared as a view type and does not require the use of a mutex lock.

The attack point of the Read Only Reentrancy vulnerability is in line 22 of the removeLiquidity function and the getSpotPriceEth function. When a user removes their liquidity assets, the function calls the call function to send the calculated amount of ETH to the msg.sender address. If the address corresponds to a contract address, its fallback function will be called to transfer the funds. At this time, the LP tokens have not been burned yet (line 24), the total liquidity of LP tokens in the pool has not changed, and the total ETH balance of the contract has decreased. The getSpotPriceEth function, which uses these two variables, will be affected. If the getSpotPriceEth function is called during a reentry attack in the fallback function of the msg.sender contract, the final result obtained will be smaller than the normal value, which means that the price of LP tokens has decreased.

There have been multiple cases of attacks on the Read Only Reentrancy vulnerability. For example, on February 9th, 2023, a hacker attacked the DeFi protocol dForcenet and made a profit of 1236 ETH and 710,000 USX tokens. On April 4th, 2023, the lending project Sentiment on Arbitrum was attacked, resulting in a loss of $1 million.

(2) Incorrect tx.origin Authorization

The difference between tx.origin and msg.sender is often overlooked in smart contract development. “tx.origin” is a global variable in the smart contract that traverses the entire call stack and returns the address that initially sent the call, which must be an external account address rather than a contract address. “msg.sender” returns the direct caller of the function, which can be either an external account address or a contract address. Using tx.origin for identity verification in a smart contract can make the contract vulnerable to phishing attacks. When a contract uses tx.origin as a condition for transfer, a malicious attacker can steal ether or tokens from the contract by constructing a designed call chain.

```
1   contract VulnerableContract {
2       address public owner;
3       constructor() {
4           owner = msg.sender;
5       }
6       function withdrawAll(address _recipient) public {
7           require(tx.origin == owner);
8           payable(_recipient).transfer(address(this).balance);
9       }
10  }
11
12  contract AttackContract {
13      VulnerableContract vulnerableContract;
14      address attacker;
15      constructor (VulnerableContract _vulnerableContract, address _attacker) {
16          vulnerableContract = _vulnerableContract;
17          attacker = _attacker;
18      }
19      receive() external payable {
20          vulnerableContract.withdrawAll(attacker);
21      }
22  }
```

Supplementary Figure 2. Example of Incorrect tx.origin Authorization

As shown in the Supplement Figure 2, there are two contracts, VulnerableContract and AttackContract. The withdrawAll function in VulnerableContract is used to withdraw all balances in the contract, and owner check is required before withdrawal (line 7). Assuming that the owner of the VulnerableContract contract calls the withdrawAll function to transfer funds to the AttackContract address (line 8), its fallback function will be called (lines 19-21). The fallback function is injected with malicious code, which calls the withdrawAll function of VulnerableContract again. In the withdrawAll function, the tx.origin obtained is the external account address that deployed VulnerableContract, so the check passes (line 7), and all balances in VulnerableContract are transferred to AttackContract. If authorization is checked using msg.sender in line 7, it will get the

AttackContract contract address instead of the external account address that deployed VulnerableContract. In this case, the check does not pass, and the transfer to AttackContract will not be executed.

(3) Timestamp Dependency

Unlike traditional solutions, the execution environment of smart contracts is on the miner's terminal. Smart contract code that includes weak randomness based on chain properties can be exploited by malicious miners. In Ethereum, timestamp is a common chain property. The timestamp of a block is the local system time of the miner who mined the block, but Ethereum allows miners to modify the timestamp of a block within a certain range (30 seconds after block validation). When certain logic in a contract depends on the current time, a miner can control the execution result by manipulating the current time, achieving a certain expectation or even gaining illegal benefits. Therefore, chain property values obtained based on "block.timestamp" have weak randomness. In addition, other fields such as "block.difficulty", "block.coinbase", and "block.number" are also insecure.

```
contract Lottery {
    uint256 private Last_Payout = 0;
    uint256 weakRanVal1 = block.timestamp;
    uint256 weakRanVal2 = block.number;
    function random() public view returns (uint256 result) {
        uint256 y = weakRanVal1 * weakRanVal2 / (weakRanVal1 % 5);
        uint256 seed = weakRanVal2 / 3 + (weakRanVal1 % 300) + Last_Payout + y;
        uint256 h = uint256(blockhash(seed));
        return uint256(h % 100) + 1;
    }
}
```

Supplementary Figure 3. Example of Timestamp Dependency

As shown in the Supplement Figure 3, it is a lottery contract. A "lucky number" is calculated based on the current block's timestamp and other variables that can be known in advance. The participant with the same encoded "lucky number" wins the prize. Miners can try different variables (such as "block.timestamp", "block.number", and "blockhash()") during mining to calculate this "lucky number" in advance, thereby controlling who can become the winner.

(4) Unhandled Exceptions

When calling low-level but important functions such as "send", "call", and "delegatecall", if an exception occurs during the call, the exception will not be propagated (reasons for exceptions in low-level calls include actively calling "revert()", insufficient gas, and call stack overflow), and only "true" or "false" will be returned, while continuing to execute the next contract instruction. If the contract code does not handle exceptions or return values for these three functions, it may cause logical errors in the code.

```
1   function withdraw(uint256 _amount) public {
2       require(balances[msg.sender] >= _amount);
3       msg.sender.send(_amount);
4       balances[msg.sender] -= _amount;
5       emit Withdraw(msg.sender, _amount);
6   }
```

Supplementary Figure 4. Example of Unhandled Exceptions

As shown in the Supplement Figure 4, the "withdraw" function is used for withdrawal operations. The code in line 3 calls the "send" function for transferring funds, but does not check the return value of the transfer. If the transfer fails, the code will still continue to execute, as shown in line 4 where the amount is deducted. This leads to the abnormal situation where the transfer fails but the balance is still deducted.
